# Exploring the use of the perceived stress scale for children as an instrument for measuring stress among children and adolescents: a scoping review

**DOI:** 10.3389/fpsyg.2024.1470448

**Published:** 2024-11-07

**Authors:** Dmitry S. Kornienko, Natalia A. Rudnova, Aleksander N. Veraksa, Margarita N. Gavrilova, Valeria A. Plotnikova

**Affiliations:** FSBSI Federal Scientific Center of Psychological and Multidisciplinary Research (FSC PMR), Moscow, Russia

**Keywords:** perceived stress scale for children, perceived stress, scale, stress assessment, children, adolescents, stress-reducing interventions, literature review

## Abstract

This review examines the application and findings related to the Perceived Stress Scale for Children (PSS-C) since its development by B. White in 2014. The PSS-C is designed to assess children’s perceived stress, focusing on their subjective experience rather than objective stressors. Our review utilized the PRISMA method to systematically collect and analyze pertinent literature, with a specific focus on studies which utilized the PSS-C. A comprehensive screening process reduced the extensive initial search results from various databases, ultimately resulting in the inclusion of 21 studies. These studies were assessed based on criteria that included publication date, language, and relevance to children’s perceived stress. We categorized the selected studies into several themes: (1) the impact of COVID-19 and the return to school; (2) mindfulness as a coping mechanism; (3) the effectiveness of breathing techniques; (4) mental health intervention programs; and (5) the cultural context of stress. The review also highlighted potential biases in the studies, particularly concerning sample size and randomization procedures. Key findings from the reviewed studies included the significant impact of the COVID-19 pandemic on children’s perceived stress, the role of mindfulness and breathing techniques in stress reduction, and the effectiveness of intervention programs. In conclusion, the review emphasized the significance of the PSS-C as a tool for evaluating perceived stress in children and stressed the necessity for additional research to examine its connections with different psychological and social factors. The findings underscore the importance of supportive parent–child interactions, especially during challenging situations such as the COVID-19 pandemic, and the possible advantages of mindfulness and other coping mechanisms in reducing stress. Subsequent research should persist in enhancing stress assessment tools and exploring the enduring impacts of stress on children’s growth and welfare.

## 1 Introduction

Stress is a fundamental concept that is crucial for understanding health and adaptability. It has evolutionary roots and includes several systems inside the body that contribute to the stress response.

The concept of stress has changed among scientists since its first definition. According to one of the most recent definitions, stress is the body’s response to any real or perceived threat, and enhances physiological and psychological activity to help cope with the stressor (McEwen and Akil, 2020). If a stimulus has the potential to trigger a stress response, we refer to it as a stressor. When the stimulus is perceived as potentially harmful, the stress response is activated.

Stress refers to not only the person’s objective physiological or psychological state, but also his or her subjective perception of stress. Cohen et al. (1983, p. 385) defined perceived stress as “the degree to which individuals perceive situations in their lives as stressful.” This concept encompasses a span of events, ranging from individual cognitive and emotional responses to potential or actual risky life events (Yılmaz Koğar and Koğar, 2024; Zolotareva, 2023).

Studies have shown a dramatic increase in perceived stress during the COVID-19 pandemic. Studies of the negative COVID-19 pandemic impact showed that among the various sources of stress, perceived stress and its emotive reactions (e.g., intrusion), impacted well-being (Fiorilli et al., 2021; Kornienko and Rudnova, 2023; Kuznetsova et al., 2021). Several characteristics, such as gender, age, work type, sport and physical activity influenced people’s perception of pandemic stress (e.g., Pervichko et al., 2022), experience of distress and propensity to post-COVID syndrome (e.g., Buonsenso et al., 2022; Di Martino et al., 2024). A cross-country comparison during the COVID-19 pandemic revealed that, while in Russia family was mentioned as the main source of the perceived stress, in Spain the negative effect was perceived to come from many sources (Ausín et al., 2020).

Because stress is one of the most influential variables for cognitive and personal development, as well as mental health and well-being, the study of children’s stress is an important area of research. The critical inquiry for child stress research pertains to the stress assessment methodology. The existing measurements of children’s stress address physiological, psychological, and observational components. There is the most consensus about measurements like cortisol or physiological stress levels (Lynch et al., 2022; Belinsky et al., 2023); however, behavioral stress measurements can be more difficult because they combine with temperament and stress reactions (Skinner and Zimmer-Gembeck, 2007). The stress of a child can be assessed using many measures for parents or caregivers, such as the “Children’s Stress Signs” scale, which consists of nine items and three categories: “psychological stress signs,” “physical stress signs,” and “positive behaviors” (Mochida et al., 2021).

Previous research on children’s perceived stress led to a broad definition and the search for indirect measurements of its assessment, but was hindered by preschoolers’ insufficient ability to distinguish between ideas and emotions (Davis and Soistmann, 2022). The theoretical concept of preschooler stress has emerged in modern research, enabling the development of empirical strategies to overcome this challenge. The finding (e.g., Lynch et al., 2022) that children are capable of providing self-reports on some stress-related events and actions throughout their preschool years has created avenues for further exploration into the stressful variables affecting children’s development.

The widely used measures for evaluating perceived stress are the scales proposed by Cohen et al. (1983; Zolotareva, 2023). Numerous countries have adapted the scale and produced short versions. Since White (2014) publication of the Perceived Stress Scale for Children (PSS-C), the problem of perceived stress among children has become the focus of a growing number of studies.

The Perceived Stress Scale for Children (PSS-C) aims to assess the perceived stress in general over the previous week without taking into account the intensity, duration, or frequency of stressors. B. White (2014) proposed that the scale is unidimensional, but some researchers have attempted to find a multidimensional structure (Takeuchi et al., 2022) or identified a two-factor structure (Kornienko et al., 2024). The scale items covered various aspects of life, such as family and peer relationships, general descriptions of state and mood, and conflicts or arguments during the previous week.

Thus, the scale better evaluates the child’s current perception of his/her environment and social relations as stressful than it does the more severe and chronic forms of stress (e.g., child maltreatment or household dysfunction), which may have a stronger influence on minors’ development and psychosocial functioning. Some argue that this instrument may have missed various forms of stress over time (Keane et al., 2023).

### 1.1 Aim of the review

The purpose of this review was to use the Preferred Reporting Items for Systematic Reviews and Meta-analysis (PRISMA) method to gather all the relevant literature and identify the main findings about the connection between the perceived stress scale for children (White, 2014) and other psychological concepts.

A special note: This literature review investigated only the stress measured with the Perceived Stress Scale for Children (White, 2014), not that which was diagnosed with other measures. The authors made this decision based on the findings of T. Lynch and colleagues’ review of research on child stress and the highlighted need for a more comprehensive understanding of children’s perceived stress (Lynch et al., 2022).

## 2 Materials and methods

### 2.1 Methods, procedures, synthesis, and screening process

Our literature review followed the guidelines of the Preferred Reporting Items for Systematic Reviews and Meta-Analysis (PRISMA). We searched for scientific studies that explored the relationship between a child’s perceived stress as measured by the Perceived Stress Scale for Children (PSS-C), and other psychological characteristics, using “perceived AND stress AND scale AND children” as the first keyword phrase, and “perceived stress scale for children” as the second.

Initially, we used Google Scholar (5 bills), PsycInfo (3), PubMed (2210), Science Direct (186) databases containing the keywords “perceived AND stress AND scale AND children.” We also addressed the Web of Science and Scopus databases, which returned a wide number of publications aligned with other databases. This search yielded results that ranged from 3 to 5 billion links, and thus prompted us to modify our search strategy to avoid redundancy and ensure a focused search. We found the best results by using Google Scholar with the keyword phrase “perceived stress scale for children,” and we accepted 151 results for further examination.

Before screening, 38 records were removed because they either (a) showed up in scientific search engines as mere citations (2); (b) were reported in languages other than English (34); or (c) they were duplicates (2).

From the abstracts that we consulted (113), 80 were eliminated because they did not meet the inclusion criteria: namely, (1) being completed and published by 2014, when The Perceived Stress Scale for Children was published; (2) being accessible for consultation directly from the site where the viewing took place, through subscriptions belonging to the Moscow State University, or by request to the corresponding authors; (3) being written in English; and (4) being empirical studies.

**Figure 1:**
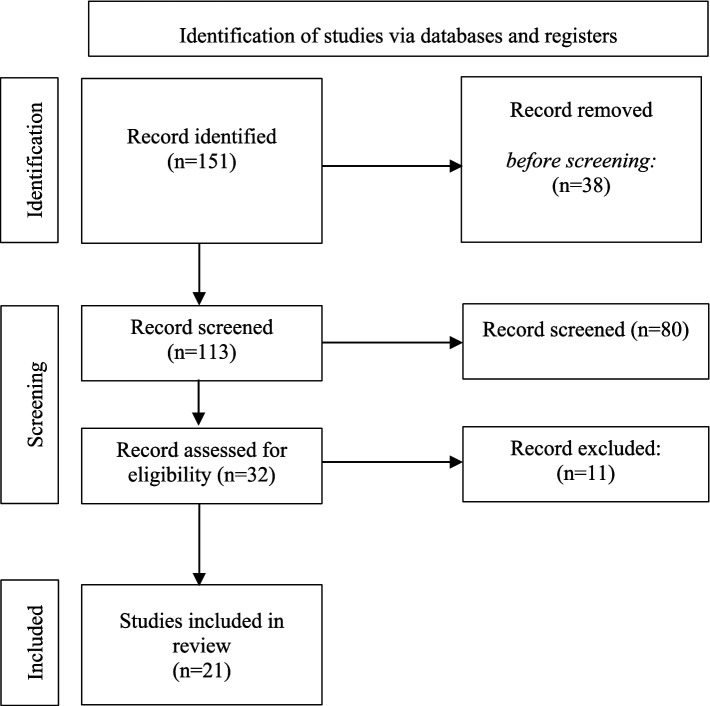
Diagram (PRISMA 2020 flow diagram) showing the flow of information through the review: the number of abstracts and articles identified, included, and excluded.

Thirty-two reports were assessed for eligibility, but among them, 11 were excluded on the basis of criteria such as (1) the use of other measurements of perceived stress; (2) not being a research article (dissertations, theses, posters, protocols, and reviews being excluded), and (3) not addressing psychological concepts. The remaining 21 studies were included in this literature review (see Figure 1).

## 3 Results

### 3.1 Description of included studies

The analysis included all studies that used the Perceived Stress Scale for Children as an assessment tool. Additionally, these studies included statistical findings about the association between perceived stress and other variables.

The studies’ brief descriptions allowed us to group them by topic: Four studies aimed to find the effects of the COVID-19 pandemic and the return to school after the pandemic restrictions were removed; three were about mindfulness as an important feature for stress coping; three were about the use of breathing techniques for lowering stress; five studies concerned the implementation of the intervention program to lower mental health problems; and the last five studies were about stress in different cultural contexts, teacher-student relationships, and risk behavior.

It should be mentioned that some topics were related, e.g., the associations between mindfulness and self-reported COVID-19 impact.

Nine of the analyzed studies used an experimental design, two included a qualitative analysis in addition to some statistics, and the remaining one primarily used a cross-sectional design.

Table 1 presents a brief description of the included studies using the Perceived Stress Scale for Children (PSS-C).

**Table 1:** Summary of observed studies using the Perceived Stress Scale for Children (PSS-C).

Authors, year, country	Article title	Sample size	Female percentage in the sample (Gender distribution)	Mean age/age distribution	Aim	Reliability of the PSS-C	Measures (except PSS-C)	Statistical main results	Risks of biases
Takeuchi et al. (2022), Japan	Identifying vulnerable children’s stress levels and coping measures during COVID-19 pandemic in Japan: a mixed method study	36	36	*M* = 11.3; range: 8–17 years	The study aimed to explore the experiences of vulnerable children in Japan during the COVID-19 pandemic, specifically focusing on those from socially disadvantaged groups		The PSS-C was modified. The scale was asking about participants’ experiences ‘during the COVID-19 pandemic’ instead of ‘in the last week’. Four composite scores were aggregated as the following variables: stressor sensitivity; emotional state; security; and time pressure.	Out of the total respondents, 16 children (53%) had moderate stress scores, and 11 children (36%) had high stress scores. The distribution of stress scores did not show any significant difference based on the children’s age or gender.	Sample size, partially completed questionnaire, children from socially disadvantaged groups.
Asemota et al. (2022), Nigeria	Exploring children’s knowledge of COVID-19 and stress levels associated with the pandemic in Nigeria: a mixed-method study.	265	52	*M* = 12.5; range 6–17 years	The goal is to evaluate children’s understanding of COVID-19 and their stress experiences during the pandemic.		Questionnaires about COVID-19, and COVID-19 prevention measures	The effect of the pandemic and its control measures on children’s mental well-being using psychological stress scales showed high stress scores. The overall mean stress score among participants was 20.47 (SD = 5.109) with the range of 3–33. The mean stress score for females was 20.59 (SD = 5.08) and for males was 20.34 (SD = 5.224).	The sample was skewed toward the middle socioeconomic class it may cause the problems of generalization to other Nigerian children. Lack of pre-pandemic data about stress.
Journault et al. (2023), Canada	Stress, anxiety, emotion regulation and social support in parent–child dyads prior to and during the onset of the COVID-19 pandemic	136	64.7	Age range 10–17 years. There are two groups of children: those aged 10–12 years old (M = 11.25, SD = 0.53); those aged 15–17 years old (M = 16.24, SD = 0.48).	To measure the change in stress and anxiety in children and parents during the onset of the COVID-19 pandemic compared to pre-pandemic	McDonald’s omega = 0.75	the State–Trait Anxiety Inventory- Revised for Adults/Children, Anxiety Sensitivity Index (adults and children), The Emotion Regulation Questionnaire for Children and Adolescents, Co-rumination questionnaire, and The Child and Adolescent Social Support Scale	The impact of the pandemic and its control measures on children’s mental well-being, as measured by psychological stress scales, revealed high levels of stress. The overall mean stress score among participants was 20.47 (SD = 5.109), with a range of 3–33. For females, the mean stress score was 20.59 (SD = 5.08) and for males, it was 20.34 (SD = 5.224).	Sample social status, participation bias due to the volunteering sampling method, sample size, the measurement of effect
Długosz (2022), Poland	Mental health disorders among students from rural areas 3 months after returning to school: A cross-sectional study among polish students	552	49	*M* = 13.2, SD =0.78; range 11–15 years.	The study’s objectives were to estimate the levels of mental health among school students and investigate the relationship between mental health indicators and higher levels of financial deprivation in students’ families.	Cronbach’s alpha = 0.806.	The WHO-5 Well-Being Index, questions regarding the identification of problems occurring during distance education	The WHO-5 well-being index negatively correlates with the perceived stress index (*r* = −0.59, *p* < 0.001). Girls have a higher level of stress (*F* = 19.18, *p* = 0.001) than boys. Students from villages achieved higher scores of perceived stress (*F* = 19.18, *p* = 0.001) and had lower school performance (*F* = 4.69, *p* = 0.01). Higher perceived stress among students is associated with lower financial status in their families (*r* = −0.10, *p* < 0.001).	Convenience sample, selection bias, self-reported measures, confirmation bias
Kanchibhotla and Subramanian (2021), India	Improvement in Children’s Mental Health and Cognitive Abilities with Yogic Breathing: A Pilot Study	420	43.6	*M* = 10.5; range 8–13 years.	The study objectives are to examine the effects of a breath-based yogic technique on children’s mental health and cognition		The WHO-5 Well-Being Index, the six-letter cancelation test (SLCT) for measuring cognitive abilities	56% of participants experienced a significant decrease in stress levels after the intervention. Participants showed significant improvement in mental well-being (lower stress level) after the yogic breathing intervention (*p* < 0.01; d = 0.152) and the follow-up effect of stress reduction (*p* < 0.01; *d* = 0.567).	Absence of a control group, potential self-selection bias, no randomization, confirmation bias.
Kanchibhotla et al. (2021), India	Association of yogic breathing with perceived stress and conception of strengths and difficulties in teenagers	455 (Experimental group = 237; control group = 218)	53 (Experimental group = 55; control group = 52)	*M* = 14; range 13–17 (Experimental group M = 14, SD = 0.77; control group *M* = 14, SD = 0.74)	The main aim of the study was to investigate the impact of a specific yogic breathing technique, on teenagers’ perceived stress levels and social behavior.		Strength and Difficulty questionnaire (SDQ)	The comparison based on gender revealed that teenagers (regardless of gender) in the experimental group had lower scores on perceived stress (*p* < 0.001) than their counterparts in the control group, indicating a positive impact of the intervention.	Selection bias, and lack of randomization, self-reported measures, confirmation bias
Angelopoulou et al. (2022), Greece	The effect of Pythagorean self-awareness on heart rate variability, perceived stress and behavior of preschool children	45	55.6	*M* = 4.12 (SD = 0.98)	The aim of this study was to examine the effect of the simplified Pythagorean Self-Awareness Intervention on heart rate variability (HRV) parameters, perceived stress, and behaviors of preschool children.		Checklist for Screening Behavioral Problems in Preschool Children, photoplethysmography (PPG) indices	The results of the second pretest and posttest showed statistically significant differences. Significant improvements were observed in perceived stress levels (*d* = 0,65; *p* < 0.001), screening for behavioral problems (*d* = 0,79; *p* < 0.001), heart rate mean (*d* = 0,70; *p* < 0.001), and low frequency/very low frequency (d = 0,32; *p* = 0.034).	Mean age, No age range, Sample size, Lack of other variable control
Maratos et al. (2024), United Kingdom	A Mixed-Methods Study of Compassionate Mind Training for Pupils (CMT-Pupils) as a School-Based Wellbeing Intervention	67		Age range 11–12 years	The study aimed to address the increasing mental health challenges in school-aged children by investigating the effectiveness of Compassionate Mind Training for Pupils (CMT-Pupils) as a school-based wellbeing intervention	Cronbach’s alpha = 0.84.	Child and Adolescent Perfectionism Scale (CAPS), Moods and Feelings Questionnaire – Short Version (MFQ-SF), Rosenberg Self-Esteem Scale (RSE), Self-Compassion Scale for Children (SCS-C), Trait Anxiety Inventory for Children (STAIC-T), Warwick-Edinburgh Mental Wellbeing Scale (WEMWBS)	The study measured the effectiveness of the Compassionate Mind Training for Pupils (CMT-Pupils) intervention on mental health levels among the participants. There was a significant time-by-group interaction effect for anxiety levels after the intervention, but not for perceived stress levels. It appears that the CMT-Pupils and control groups felt the same stress levels after the intervention.	Non-random group assignment, Potential social desirability bias due to teacher presence during intervention, No correction for multiple comparisons in quantitative analyses, increasing risk of type 1 errors, Potential underpowering, increasing risk of type 2 errors
Treves et al. (2023a), USA	Mindfulness supports emotional resilience in children during the COVID-19 pandemic	163	53.4	Age range 8.2–10.6 years, SD = 6 months	The study aims to investigate whether trait mindfulness moderates the relationship between self-reported COVID-19 impact and its negative association with measures of anxiety, sadness, stress, and negative affect in children.	Cronbach’s alpha = 0.62.	Child COVID Impact Scale, Revised Child Anxiety and Depression Scale (RCADS-25-C), Negative Affect, Perceived Stress Scale for Children (PSS-C), Child and Adolescent Mindfulness Measure (CAMM)	Greater trait mindfulness in children was associated with less stress (*r* = −0.04, *p* < 0.0001). Trait mindfulness moderated the relationship between children’s perceived impact of COVID-19 and their negative affect, such that higher mindfulness was associated with no correlation between COVID-19 impact and negative affect.	Selection bias, unstandardized measures, cross-sectional design
Treves et al. (2023b), USA	At-home use of app-based mindfulness for children: A randomized active-controlled trial	314	43.7	*M* = 9.38, SD =0.54	To investigate the effectiveness of a remote app-based mindfulness intervention for promoting children’s wellbeing.	Cronbach’s alpha = 0.87, McDonald’s omega = 0.91	Child self-reported measures: anxiety and depression symptoms (RCADS-25-C), negative affect, trait mindfulness (CAMM). Parent-reported measures: child negative affect, child prosociality, child executive functioning (BRIEF). Parent self-reported measures: parental stress.	The intervention led to larger decreases in child-perceived stress compared to the control groups [*t* (148.74) = 1.79, *p* = 0.038], but ingroup change was greater than between groups.	Participants recruitment procedure, small to moderated effect between interventions, participants social status, self-report measures.
Sobko et al. (2020), Hong Kong	Impact of outdoor nature-related activities on gut microbiota, fecal serotonin, and perceived stress in preschool children: the Play&Grow randomized controlled …	55 (Experimental group = 30; control group = 24)	Experimental group = 52; control group = 44; n.s.	Age range 2–5 years. Experimental group *M* = 2.98, SD = 1.02; control group *M* = 2.95, SD = 0.68	The study objective is to investigate the impact of the intervention program on the biological characteristics and psychological well-being of 2–5-year-old children		Children’s psychosocial measurement, connectedness to nature, serotonin measurement, and gut microbiota analysis	Compared to the control group, the overall score on the perceived stress scale for children (PSS-C) significantly improved post-intervention (*p* = 0.05).	Sample size, lack of biological analysis details, selective display of results with *p*-value 0.05
Patil et al. (2021), India	Effectiveness of art therapy on level of stress and anxiety among pediatrics oncology patients	30 (Experimental group = 15; control group = 15)	40	Age range 7–12 years	The study aims to assess the effectiveness of art therapy on stress and anxiety levels among pediatric oncology patients.		Structured questionnaire for demographic data, Hamilton anxiety rating scale (HAM-A)	The experimental group that received art therapy showed statistically significant reductions (p < 0.001) in stress scores among children with cancer compared to the control group. However, the between-group comparison revealed no significant differences in perceived stress levels after the intervention.	Sample size, lack of generalizability, self-reported measures, confirmation bias.
Rongo et al. (2024), Italy	Evaluation of psychosocial aspects in patients with juvenile idiopathic arthritis	73	76.7	Age range 6–16 years	The study’s goal is to clarify the relationship between TMJ (temporomandibular joint) signs and symptoms, psychosocial factors, and broader clinical manifestations in JIA (juvenile idiopathic arthritis) patients.		Physical function, Measurement of Pain Intensity, Disease assessment, Assessment of Health-Related Quality of Life (HRQoL), Overall Well-being Evaluation, The Pain Catastrophizing Scale, The Revised Child Anxiety and Depression Scale-Short Version (RCADS-SV), the Orofacial Pain Questionnaire, the Jaw Functional Limitation Scale (JFLS-8)	There is a strong link between perceived stress and anxiety (*r* = 0.507, *p* < 0.001), depression (*r* = 0.610, *p* < 0.001), pain catastrophizing (*r* = 0.287, *p* = 0.014), disease activity (*r* = 0.251, *p* = 0.032), general health status score (*r* = 0.534, *p* < 0.001), jaw function limitations (*r* = 0.306, *p* = 0.009), and the juvenile arthritis multidimensional assessment (JAMAR) score (*r* = 0.455, *p* < 0.001). The regression model for perceived stress has significant predictors: disease activity (*b* = 0.1, *p* = 0.03), general health status score (*b* = 0.44, *p* < 0.001), JAMAR Total Symptoms Score (*b* = 0.17, *p* = 0.002), JAMAR Total Score (*b* = 0.97, p < 0.001), and impact of disease on quality of life (*b* = 0.14, *p* = 0.003).	Sample bias, Cross-sectional design, Lack of a non-JIA control group
Vasanthakumari et al. (2016), India	Effectiveness of stress reduction technique on the level of stress among HIV infected children.	60 (Experimental group = 30; control group = 30)	No information	Age range 8–15 years	The objective of the study was to assess the effectiveness of a stress reduction technique on the level of stress among HIV infected children.		Demographic variables, the Perceived Stress Scale for Children (PSS-C)	A significant reduction in perceived stress levels was found among the HIV-infected children in the experimental group after implementing the stress reduction technique (*t* = 4.190, *p* < 0.001). This robust statistical significance underscored the effectiveness of the intervention in alleviating stress among the experimental group compared to the control group (*t* = 3.820, *p* < 0.001).	Sample size, lack of generalizability, confirmation bias, Lack of other variable control
Journault et al. (2024), Canada	Learning to embrace one’s stress: the selective effects of short videos on youth’s stress mindsets	458 (Experimental group = 233; control group = 225)	83	Age range 14–17 years.	The study intended to evaluate the potential rapid effects of the intervention on stress mindsets (perceived stress and anxiety sensitivity) in adolescents through a comparison of outcomes between the intervention group exposed to Stress N′ Go videos and the control group exposed to Brain Facts videos.	Cronbach’s alpha: pre-intervention = 0 0.67, post-intervention = 0.71	the Stress Mindset Measure-General (SMM-G), Childhood Anxiety Sensitivity Index (CASI)	The results indicated a non-significant time-condition interaction effect on perceived stress, with an *F*-value of 0.02 and a *p*-value of 0.899. This suggests that neither the intervention condition nor time had a significant influence on participants’ perceived stress levels. By using the BIC of null and alternative models, the study found a Bayes factor of 31.50 for perceived stress. This supported the conclusion that the intervention did not have a significant effect on perceived stress among the adolescents.	Sample bias, High attrition rate, Potential selection bias, Lack of validation of the stress mindset measure
Jones et al. (2023), Switzerland	Stress, mental health and sociocultural adjustment in third culture kids: exploring the mediating roles of resilience and family functioning	143	60	Age range 7–17 years	The study’s objectives were to investigate how perceived stress and acculturative stress affect the adjustment of TCKs (“Third Culture Kids”) in terms of mental health difficulties and sociocultural adjustment, and to determine whether resilience and family functioning mediate the relationships between stress predictors and adjustment outcomes.	Cronbach’s alpha = 0.82.	Acculturative Stress Inventory for Children (ASIC), Child and Youth Resilience Measure (CYRM-12), Strength and Difficulties Questionnaire (SDQ), McMaster Family Assessment Device, Sociocultural Adaptation Scale (SCAS-Child)	Perceived stress negatively correlates with resilience (*r* = −0.56, *p* < 0.05) and positively with difficult sociocultural adaptation (*r* = 0.52, *p* < 0.01). The effect of age on perceived stress was significant (*b* = 7.99, *p* < 0.001). Resilience mediates the relationship between perceived stress and mental health difficulties (*b* = 0.15, *p* < 0.05), suggesting that building resilience could improve mental health outcomes for TCKs.	The study was conducted during the COVID-19 pandemic, which likely increased stress for the participants. Selection bias, the sample had limited contextual variation.
Keane et al. (2023), USA	Teacher–student relationships, stress, and psychosocial functioning during early adolescence	288	54	*M* = 12.01; range 11.1–13.7 years	The study aims to investigate the connections between teachers and students, their psychosocial development in the early stages of adolescence, and the influence of teacher-student conflict and intimacy on this development.	Cronbach’s alpha = 0.69	the Achenbach System of Empirically Based Assessment (ASEBA) Youth Self-Report (YSR), the Student–Teacher Relationship Scale (STRS) short-form,	Higher perceived stress is positively related to student-teacher conflict (*r* = 0.24, *p* < 0.01), anxiety (*r* = 0.38, *p* < 0.01), aggressive behavior (*r* = 0.27, *p* < 0.01), and rule-breaking (*r* = 0.32, *p* < 0.01). Perceived stress is the main significant predictor for anxiety (*b* = 0.49, *p* < 0.01), aggressive behavior (*b* = 0.34, *p* < 0.01), and rule-breaking (*b* = 0.28, *p* < 0.01), but those effects disappear over time.	Sample bias, perceived stress measure sensitivity, low internal consistency of measures, sample attrition
Manns et al. (2022), USA	Perceived Stress and Nonverbal Intellectual Abilities Are Differentially Related to Academic Success in Latinx and European American Rural Elementary Students	44 [Latinx (*n* = 13; 28%) and European American (EA) students (*n* = 31; 66%)]	Latinx female = 46.15%; EA female = 48.39%;	Latinx *M* = 8.86, SD = 0.61; EA *M* = 9.00, SD = 0.52; *t*(42) = 0.78, *p* = 0.44	This study explores how stress, cognitive abilities, and academic performance are linked in Latinx and European American rural elementary students		The Wechsler Nonverbal Scale of Intelligence, statewide academic testing results	Latinx and European American elementary students reported similar levels of perceived stress. For Latinx students, better coding performance was linked to lower stress levels (*r* = −0.67; *p* < 0.05), and for European American students, higher spatial span abilities correlated with lower perceived stress (*r* = −0.40; *p* < 0.05).	Sample size, unbalanced sample, bilinguality of Latinx subsample, social status of participants. Only correlation findings. No age range.
Hodge et al. (2023), Puerto Rico	Puerto Rican pre-teenagers’ physical and sedentary activities, dietary trends, and stress	107	41	*M* = 8.7, SD = 1.2; range 6–11 years	The study aim to identify and analyze preadolescents’ perceived stress, weight status, physical and sedentary activity tendencies, and dietary behavior in Puerto Rico.		Physical activity, sedentary activity, dietary intake, and patterns were diagnosed with surveys, height, and weight measurement	The descriptive statistics of the items on the PSS-C indicate that the participants generally had or expressed low to modest levels of stress. The participants only sometimes worried about their grades or school (*M* = 2.6) and about having enough time to do what they wanted (*M* = 2.8).	Self-reported data on physical activity and sedentary behavior, the lack cross-cultural validity and reliability of surveys, gender bias, convenience sample
Beranbaum et al. (2023), South Africa	Behavioral and biological indicators of risk and well-being in a sample of South African youth	83	Both genders, no additional details	*M* = 10.69, SD = 0.85; range 9–13 years.	Using a multi-method assessment that includes heart rate variability, a risk-taking task, and self-report measures, the study aim to understand the risk and resilience factors for youth who have either witnessed or directly experienced violence.	Cronbach’s alpha = 0.52.	Risk-taking propensity (BART_Y), self-esteem (IAT), heart rate variability (emWave ear sensor), exposure to violence (CEO), sensation seeking (BSSS-C)	There was no correlation between PSS and age [*r*(81) = −0.01, *p* = 0.91] or gender [*r*(81) = 0.21, *p* = 0.06]. The study found a negative correlation between risk-taking propensity and PSS (*r* = −0.29, *p* = 0.015), suggesting that participants who endorsed more stress demonstrated less risk on the risk-taking behavioral task. The relationship between PSS and HRV was not significant (*r* = 0.22, *p* = 0.07).	Sample size, lack of demographic information, low internal reliability of some self-report questionnaires, reliance on norms from other countries
Marie et al. (2022), Canada, Australia	A Cross-Sectional Study Investigating Canadian and Australian Adolescents’ Perceived Experiences of COVID-19: Gender Differences and Mental Health Implications.	1,326	71% female overall (78% Canadian sample, 57% Australian sample)	*M* = 15.36, SD = 1.23; range 13–18 years	The aim of this study is to investigate the differences between Canadian and Australian adolescents in their subjective experiences of COVID-19, as well as the gender differences in these experiences.	Cronbach’s alpha: Canada = 0.71; Australia = 0.80	COVID-19 questionnaire, the Stress Mindset Measure—General (SMM- G), The Childhood Anxiety Sensitivity Index (CASI), STAIC-S, CTAS, the Co-Rumination Questionnaire (CRQ), The Beck Depression Inventory-II (BDI-II), the Patient Health Questionnaire-9 for Adolescents (PHQ-9A)	Canadian adolescents reported experiencing significantly more stress related to COVID-19 compared to Australian adolescents. Both Canadian and Australian girls reported significantly higher levels of stress related to COVID-19, and they also displayed significantly more signs of depression.	Self-report measures, The COVID-19 questionnaire was not a validated scale/Most of the reported differences were small. Cross-sectional design. Potential confounding due to higher proportion of females in Canadian sample Selection bias.

### 3.2 Country of publication of the included studies

Seven studies from North and Central America, five from Europe, four from India, two from Africa, and two from Asia made up the geographically distributed research in the literature review. It is important to note that the study only included papers published in English.

### 3.3 Years of publication of the included studies

With one exception, all of the included studies were published within the previous 5 years.

### 3.4 Mean ages of the samples in the included studies

Participants in the studies included in this review ranged in age from 2 to 17 years, with a mean age of 9.27. In most studies, school students made up the majority of the samples. Four studies reported the relationship between age and perceived stress (Beranbaum et al., 2023; Długosz, 2022; Jones et al., 2023; Takeuchi et al., 2022), but only two of them present statistical evidence. Only one study reported age as a significant predictor of perceived stress (Jones et al., 2023). The general result was that perceived stress was unrelated to the participants’ ages.

### 3.5 Social status

The description of the study’s limitations or participant descriptions indicated that the participants’ social status ranged from extremely low to middle and high socioeconomic status. Only one study presented a comparison of the participants with different indicators of social status (Długosz, 2022). They found that students from villages had higher scores of perceived stress (*F* = 19.18, *p* = 0.001) and lower school performance (*F* = 4.69, *p* = 0.01). Higher perceived stress among students was associated with lower financial status in their families (*r* = −0.10, *p* < 0.001) (Długosz, 2022).

### 3.6 Gender distributions of the samples in the included studies

The samples exhibit a range of sex distributions, with females comprising anywhere from 36 to 83% of the population. Only seven studies had an approximately equal distribution of sexes, with females accounting for 52–55%. The remaining studies had varying percentages of females: less than 50% in nine studies, two studies with percentages falling between 60 and 70%, and two studies with percentages ranging from 70 to 80%.

Seven studies reported the perceived stress score based on gender, but only three showed statistical evidence of a gender difference (Długosz, 2022; Journault et al., 2023; Marie et al., 2022); the remaining studies suggested no gender differences (e.g., Beranbaum et al., 2023).

In summary, we may speculate that girls had or had reported higher stress, but future studies need to clarify this finding.

### 3.7 Reliability of the perceived stress scale

The majority of studies used the Perceived Stress Scale for Children (PSS-C), which comprises 14 items. Each item can be answered on a Likert scale consisting of four options (never—a little—sometimes—a lot). The perceived stress score is obtained by summing the responses to all items (the first question is not included in the score).

A higher value on the total score suggests higher stress perceptions and questions. Feeling rushed or worried about not having enough time for a desired activity, the pressure of academic performance, the poor quality of friendships, and poor relationships with parents all contributed to stress perception. This scale assesses the extent to which individuals feel that their lives have been unpredictable, uncontrollable, and overloaded over the past weeks.

Ten studies reported the reliability of the PSS-C; Cronbach’s alpha varied from 0.52 to 0.84 with a mean of 0.73; two studies additionally reported McDonald’s omega (Journault et al., 2023; Treves et al., 2023b). In most studies, the scale’s reliability was acceptable. Only one study (Beranbaum et al., 2023) excluded the PSS-C results due to their low reliability.

Just one study used a modified version of the Perceived Stress Scale for Children, which consists of 8 items with a Likert scale from 5 (very often) to 0 (never). A higher total value on all questions corresponded to a higher stress perception. The alpha coefficient for that version was 0.81 (Długosz, 2022).

Two studies (Asemota et al., 2022; Takeuchi et al., 2022) modified the perceived stress scale for children (PSS-C) in response to the COVID-19 pandemic. In those studies, the participants were asked about their experiences “during the COVID-19 pandemic” instead of “in the last week.” In addition, those studies (Takeuchi et al., 2022) proposed new subscales for the PSS-C: stressor sensitivity, emotional state, security, and time pressure. These composite scores may be of interest for future studies.

### 3.8 Risk of bias in included studies

**Figure 2:**
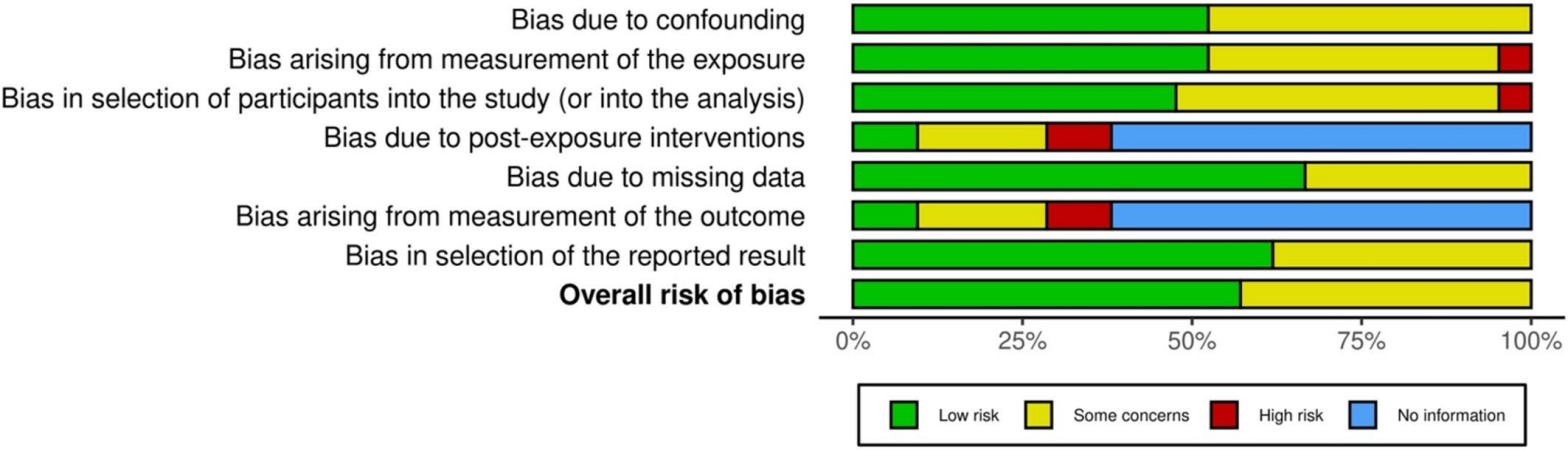
The risk of Bias Graph. The risk of bias graph presents ratings for all studies included in the review.

The methodological quality of the studies varied due to differences in design, sample populations, and objectives. Bias ratings were generally split between ‘Low’ and ‘Some concerns,’ with confounding and measurement biases both nearly evenly divided (52 and 48%, respectively). Selection bias also showed a similar pattern (48% ‘Low,’ 48% ‘Some concerns’), while one study (5%) was rated ‘High’ risk due to lacking a control group. Four of the included studies reported a power analysis to determine a sample size calculation prior to the intervention or data collection (Maratos et al., 2024; Treves et al., 2023b). Among the reviewed studies, nine acknowledged a sample size of less than 100 participants. In three cases, the size of the sample was explained by the group’s uniqueness: HIV-infected children (Vasanthakumari et al., 2016), patients with juvenile idiopathic arthritis (Rongo et al., 2024), and children attending three non-profit organizations that provide support for groups experiencing social disadvantage (Takeuchi et al., 2022). One study, along with a sample of children, also included the children’s parents (Journault et al., 2023). For studies with experimental designs (38%), 10% were rated ‘High’ risk, 19% ‘Some concerns,’ and 10% ‘Low’ in terms of intervention bias. Three studies (Maratos et al., 2024; Sobko et al., 2020; Treves et al., 2023b) detailed the randomization of participants into groups and the randomization procedures. The other publications did not include any randomization. Missing data bias was mostly ‘Low’ (67%), while selective reporting bias was primarily ‘Low’ (62%). In the ‘Risk of bias from outcome measurement,’ 10% were ‘Low’ risk, 19% ‘Some concerns,’ and 10% ‘High,’ though 62% had insufficient information. Finally, reporting bias was predominantly ‘Low’ (62%), with 38% showing ‘Some concerns.’ (see Figure 2).

All the studies included in the analysis were susceptible to bias because of the difficulties in concealing the identities of participants, their families, and caregivers throughout the implementation of psychosocial interventions.

## 4 Discussion

Previous studies had not aimed to investigate the associations of the perceived stress measurements on the Perceived Stress Scale for Children (PSS-C) (White, 2014) with children’s biological and psychological features, resulting in a knowledge gap. The purpose of current review was to scrutinize the application of the Perceived Stress Scale for Children in studies carried out over the past decade, along with the ensuing discoveries. The observed articles primarily examined five key areas: the influence of COVID-19 on children’s perceived stress levels; the significance of mindfulness traits in mitigating perceived stress levels; the beneficial effects of breathing techniques; the effectiveness of different intervention programs in reducing perceived stress levels; and the investigation of perceived stress in diverse contexts, encompassing both healthy and unhealthy children and children from various cultures. The following paragraphs summarize those topics.

### 4.1 The COVID-19 impact on the perceived stress level among children

Five studies discussed the pandemic’s impact on perceived stress and other psychological characteristics. Two with the same approach stand out. The same researchers conducted these studies using similar methods (Asemota et al., 2022; Takeuchi et al., 2022). They used the perceived stress scale for children (PSS-C) in conjunction with questionnaires about COVID-19 and preventive measures. Both studies conducted quantitative analyses of each item on the perceived stress scale for children (PSS-C) and described the main general and home stressors, such as disruptions to schooling or pandemic restrictions, protective factors, and the quality of family and friends’ relationships. As for the qualitative data, one study reported the percentage of participants who had a moderate (53%) or high (11%) stress score with no gender or age differences (Takeuchi et al., 2022). Asemota et al. (2022) presented only the mean stress score and mean scores for genders, without making any statistical comparison.

In line with the importance of parent–child interactions as a supportive factor, another study dealt with that question (Journault et al., 2023). The main result was that during the onset of the COVID-19 pandemic, parents and children responded differently in terms of stress, anxiety, and emotion regulation strategies. A more comprehensive view revealed that children exhibited equivalent levels of stress during the pandemic as they did prior to it. Perceived stress did not differ significantly across three time points in these children but girls reported higher stress than boys [*F*(1, 114) = 10.15, *p* = 0.002, η2p = 0.08] (Journault et al., 2023).

A cross-sectional study among Polish students aimed to examine the relationship between mental health indicators after post-pandemic returning to school and level of financial deprivation in students’ families (Długosz, 2022). This study was done 3 months after the children returned to school after the COVID-19 pandemic. The student’s mental health indicators, specifically perceived stress and depression were assessed using the Perceived Stress Scale for Children (PSS-C) and five items from the WHO-5 Well-Being Index, respectively. Using a series of questions, the researcher assessed the issues that arise during distance education. Sixty-four percent of the surveyed students still indicated a moderate overall level of perceived stress (*M* = 16.1; SD = 5.92). The PSS-C item-level analysis indicated that students had a moderate to high level of stress upon returning to school following distant learning. The WHO-5 well-being index negatively correlated with the PSS-C index (*r* = −0.59, *p* < 0.001), leading to the conclusion that the higher well-being (low depression) is linked with higher perceived stress. In addition, girls (aged 11–15) reported a significantly higher level of perceived stress [*F* (1, 503) = 19.18; *p* < 0.001] (Długosz, 2022). The general conclusion was that returning to school does not have a positive impact on the students’ mental health.

The comparison of the Canadian and Australian adolescents’ perceived stress during the COVID-19 pandemic showed significant differences. Australian adolescents reported experiencing significantly more perceived stress than Canadian adolescents [*F*(1, 1,179) = 8.438, *p* = 0.004, n2 = 0.007], with the exception of the stress factors of feeling angry and social restrictions. Moreover, girls in both groups reported experiencing significantly higher perceived stress than boys [*F*(1, 1,179) = 40.105, *p* < 0.001, n2 = 0.033] (Marie et al., 2022).

### 4.2 Perceived stress and mindfulness

Three studies paid attention to trait mindfulness and its associations with features of the participants’ personality, emotional characteristics, and social-behavioral features. The first correlational study aimed to investigate whether trait mindfulness moderated the relationship between self-reported COVID-19 impact and negative affect in children (Treves et al., 2023a). Along the evaluation of perceived stress (PSS-C), there were measures of mindfulness [Child and Adolescent Mindfulness Measure (CAMM), anxiety, depression (Revised Child Anxiety and Depression Scale (RCADS-25-C), and negative affect)]. The main result showed that mindfulness did moderate the relationship between COVID-19 child impact and negative affect. Increased levels of trait mindfulness may have assisted children in effectively managing a diverse array of COVID-19-related stresses including perceived stress. Greater trait mindfulness in children was associated with less perceived stress (*r* = −0.40, *p* < 0.0001), suggesting that trait mindfulness cloud allows youngsters to better manage a range of stressors (Treves et al., 2023a).

A randomized controlled trial with two control groups studied the effect of the remote application-based 8-week mindfulness intervention on promoting children’s well-being. Through parental reports, the mindfulness app revealed evidence of the intervention reducing self-perceived stress (measured with PSS-C) and negative affect in children. However, it did not significantly decrease symptoms of anxiety or depression (Treves et al., 2023b). The intervention led to larger decreases in child-perceived stress compared to the control groups [*t* (148.74) = 1.79, *p* = 0.038], but within-group change was greater than between the groups.

The other study investigated the effectiveness of compassionate mind training for pupils (CMT-Pupils) as a school-based intervention for adolescent well-being (Maratos et al., 2024). Along with the perceived stress level evaluation (with the PSS-C), researchers used measures of anxiety, self-esteem, self-compassion, and perfectionism. There was a significant time-by-group interaction effect for anxiety levels after the intervention, but not for perceived stress levels.

### 4.3 Perceived stress and breathing techniques

Three studies could be merged into one topic because they were about breathing techniques as an intervention to improve mental health, cognitive abilities, and lower perceived stress and behavioral problems (Angelopoulou et al., 2022; Kanchibhotla and Subramanian, 2021; Kanchibhotla et al., 2021). The first study about the effect of the simplified Pythagorean Self-Awareness Intervention (PSAI) on heart rate variability (HRV) parameters, perceived stress, and preschool children’s behaviors used the following measures: the Perceived Stress Scale for Children (PSS-C), the Checklist for Screening Behavioral Problems in Preschool Children and the photoplethysmographic (PPG) indices (Angelopoulou et al., 2022). There were large significant differences between the PSAI group and the control group in how stressed children felt (*d* = 0.65; *p* < 0.001); how often they exhibited behavioral problems (*d* = 0.79; *p* < 0.001); how often their heart rate went up and down (*d* = 0.70; *p* < 0.001); and how often they heard low or very low frequencies (*d* = 0.32; *p* = 0.034) (Angelopoulou et al., 2022).

Two other studies aimed to investigate the effects of a breath-based yogic technique on children’s mental health and cognition, as well as its long-term impact (Kanchibhotla and Subramanian, 2021; Kanchibhotla et al., 2021). They used the WHO-5 Well-Being Index for general mental health evaluation, the six-letter cancelation test (SLCT) for measuring cognitive abilities, and the Strength and Difficulty Questionnaire (SDQ) for assessment of the social behavoir. The Perceived Stress Scale for Children (PSS-C) measures perceived stress as one of the main outcomes. There was a positive effect of the intervention; 56% of participants experienced a significant decrease in stress levels. People who did the yogic breathing exercise had a significant improvement in their mental health (lower stress level) after the intervention (*p* < 0.01; *d* = 0.152), and this improvement continued after the intervention (*p* < 0.01; *d* = 0.567) (Kanchibhotla and Subramanian, 2021). Between-group comparison revealed that teenagers (regardless of gender) in the experimental group had lower scores on perceived stress (*p* < 0.001) than their counterparts in the control group, indicating a positive impact of intervention (Kanchibhotla et al., 2021).

### 4.4 Perceived stress among children with health problems

We grouped five studies because they all examined the impact of intervention programs on various groups of children, including some with clinical conditions.

In one study, the researchers wanted to find out how TMJ (temporomandibular joint) symptoms and signs related to other health problems in kids with juvenile idiopathic arthritis. Stress is significantly linked to chronic illnesses such as juvenile idiopathic arthritis, adversely affecting quality of life and overall well-being (Rongo et al., 2024). The Perceived Stress Scale for Children (PSS-C) measures perceived stress as one of the main outcomes. There are also a variety of measures used, including anxiety, pain, and depression scales, health quality and well-being scales, and several orthodontic questionnaires. There was a strong link between perceived stress and anxiety (*r* = 0.507, *p* < 0.001), depression (*r* = 0.610, *p* < 0.001), pain catastrophizing (*r* = 0.287, *p* = 0.014), disease activity (*r* = 0.251, *p* = 0.032), general health status score (*r* = 0.534, *p* < 0.001), jaw function limitations (*r* = 0.306, *p* = 0.009), and the juvenile arthritis multidimensional assessment (JAMAR) score (*r* = 0.455, *p* < 0.001). The regression model for perceived stress had significant predictors: disease activity (*b* = 0.1, *p* = 0.03); general health status score (*b* = 0.44, *p* < 0.001); JAMAR Total Symptoms Score (*b* = 0.17, *p* = 0.002); JAMAR Total Score (*b* = 0.97, *p* < 0.001); and the impact of the disease on quality of life (*b* = 0.14, *p* = 0.003). However, perceived stress did not reveal a significant predictor of disease’s impact on quality of life. There was no difference in perceived stress between groups with orofacial signs and symptoms and their counterparts.

The other four studies looked at how interventions affected the physical and mental health of three groups of children: healthy children (Journault et al., 2024; Sobko et al., 2020), children who were pediatric oncology patients (Patil et al., 2021); and children with HIV (Vasanthakumari et al., 2016). All three studies utilized the Perceived Stress Scale for Children (PSS-C) to assess stress and as a primary outcome measure. The interventions included the educational ecological program, videos about embracing stress, art therapy, and breathing and relaxation techniques. All these intervention programs had a significant effect (from *p* < 0.05 to *p* < 0.001) on perceived stress.

For example, a significant reduction in perceived stress levels was found among the HIV-infected children in the experimental group after the application of the stress reduction technique (*t* = 4.190, *p* < 0.001), as well as in between-group comparison (*t* = 3.820, *p* < 0.001) (Vasanthakumari et al., 2016). The exception to this was a study conducted by Journault et al. (2024), which examined the potential immediate effects of an intervention on stress mindsets in adolescents through the use of stress-reduction videos. The results indicated a non-significant effect on perceived stress.

### 4.5 Perceived stress in different cultural context

Five other studies had different objectives, but they examined perceived stress in different countries and involved participants with different cultural backgrounds.

A study about the cultural adaptation of preadolescents and teenagers due to migration was carried out in Switzerland (Jones et al., 2023). It revealed that perceived stress was a key factor that influenced mental health and sociocultural adjustment. Perceived stress [measured with the Perceived Stress Scale for Children (PSS-C)] negatively correlated with resilience (*r* = −0.56, *p* < 0.05) and positively with difficult sociocultural adaptation (*r* = 0.52, *p* < 0.01). Resilience mediated the relationship between perceived stress and mental health difficulties (*b* = 0.15, *p* < 0.05), suggesting that building resilience could improve mental health outcomes for children (Jones et al., 2023).

Groups of Latinx and European-American elementary students in rural regions of the United States underwent cross-cultural comparisons in perceived stress, cognitive abilities, and academic performance. The following measures were used — the Perceived Stress Scale for Children (PSS-C), the Wechsler Nonverbal Scale of Ability (WNV), and the Oregon Assessment of Knowledge and Skills (OAKS). While both groups reported similar levels of perceived stress, the associations between stress and cognitive abilities revealed differences. For Latinx students, better coding performance was linked to lower stress levels (*r* = −0.67; *p* < 0.05). European-American students’ higher spatial span abilities were related to lower perceived stress (*r* = −0.40; *p* < 0.05) (Manns et al., 2022).

The other study found that Puerto Rican preadolescents showed a moderate to low level of perceived stress (measured with the PSS-C), which the authors explained by the positive social and economic growth in Puerto Rico since 2017. Among the perceived stress factors, preadolescents mostly worried about their school grades and about having enough time to do what they wanted (Hodge et al., 2023). School-related problems were also discussed in the study of the role of teacher-student conflict and closedness on psychosocial functioning during adolescence (Keane et al., 2023). The Perceived Stress Scale for Children (PSS-C), the Achenbach System of Empirically Based Assessment (ASEBA) Youth Self-Report (YSR) and the Student-Teacher Relationship Scale (STRS) were used as measures. It was found that perceived stress was the main significant predictor of anxiety (*b* = 0.49, *p* < 0.01), aggressive behavior (*b* = 0.34, *p* < 0.01), and rule-breaking (*b* = 0.28, *p* < 0.01) among adolescents, but those effects disappear over the course of adolescence (Keane et al., 2023). A South African study’s results revealed a negative correlation between risk-taking propensity and perceived stress (PSS-C) (*r* = −0.29, *p* = 0.015) among adolescents, but no relationship with heart-rate indicators (Beranbaum et al., 2023).

### 4.6 Review limitations

This review was the first to describe and identify the breadth of evidence of stress among children of different age groups using the Perceived Stress Scale For Children (PSS-C) (White, 2014). Note that the methodology, with its tight inclusion criteria and search strategy, facilitated the comprehensive identification of studies. Several factors limit the conclusions that can be drawn from this systematic review. The most important factor is the quality of the included studies. The grading of many papers indicated a serious risk of bias and low overall quality. Many of the papers with a small sample size may have limited ability to demonstrate statistical significance, creating the possibility for the results to appear poorly correlated when, in fact, this may be due to poor study design. We subjectively created the possible topic groups for the reviewed studies, but they could change because of the intersecting aims and study variables.

### 4.7 Future work

A future study should focus on a narrower topic in order to undertake a comprehensive analysis, especially the perceived stress levels (evaluated with the Perceived Stress Scale for Children (PSS-C)) among adolescents with health conditions. An alternative approach would include evaluating many indicators of perceived stress in children to determine the advantages and disadvantages of these stress measures. This study has examined several techniques aimed at reducing perceived stress. However, it is important to note that the list of studies is not comprehensive, and future research should strive to find other viable interventions and methodologies. Finally, it is necessary to conduct research to determine whether the Perceived Stress Scale for Children (PSS-C) yields a single overarching variable for measuring perceived stress, or whether additional feasible subscales should be considered.

## 5 Conclusion

The current review provides a holistic view concerning the main associations between perceived stress, measured with the Perceived Stress Scale for Children (PSS-C) and other psychological characteristics among children. The following summarizes our findings about children’s perceived stress.

1. Despite the pandemic, most schoolchildren reported a moderate stress level without significant fluctuation during the observed period, according to studies about perceived stress during or after the COVID-19 pandemic.
2. The reviewed studies confirmed the negative association between mindfulness and perceived stress. One could treat mindfulness more as a protective or preventive factor for anxiety than for perceived stress.
3. Breathing techniques, used as a tool to reduce perceived stress, have a significant impact. Regular implementation of breathing techniques resulted in a decrease in psychophysiological and psychological features, including perceived stress, leading to a positive state of well-being.
4. The results of intervention programs, which targeted both healthy and unhealthy groups of children, were ambiguous. Stress reduction interventions and therapy might have a positive effect on special groups, but the generalization is questionable.
5. The manifestations of perceived stress varied due to various biological and social factors. Many studies reported a higher level of stress among girls despite their age, as well as a relationship between perceived stress and heart rate variables. A higher perceived stress level is associated with low social status, and the socio-economic status of families significantly influenced the ability to cope with stress.

## Data Availability

The original contributions presented in the study are included in the article/supplementary material, further inquiries can be directed to the corresponding author/s.

## References

[ref1] Angelopoulou K. Zaverdinou E. Bacopoulou F. Chrousos G. P. Giannakakis G. Kanaka-Gantenbein C. . (2022). The effect of Pythagorean self-awareness on heart rate variability, perceived stress and behavior of preschool children. Children 9:1529. doi: 10.3390/children9101529, PMID: 36291465 PMC9600468

[ref2] Asemota O. A. Napier-Raman S. Takeuchi H. Raman S. Asemota E. A. Nonye E. (2022). Exploring children’s knowledge of COVID-19 and stress levels associated with the pandemic in Nigeria: a mixed-method study. BMJ Paediatrics Open 6:e001444. doi: 10.1136/bmjpo-2022-001444, PMID: 36053587 PMC9226462

[ref3] Ausín B. Castellanos M. A. González-Sanguino C. Vakhantseva O. V. Almazova O. V. Shaigerova L. A. . (2020). Psychological impact of six weeks of lockdown as a consequence of COVID-19 and the importance of social support: a cross-cultural study comparing Spanish and Russian populations. Psychol. Russia 13, 89–105. doi: 10.11621/pir.2020.0406

[ref4] Belinsky A. V. Devishvili V. M. Chernorisov A. M. Lobin М. А. (2023). Hardware-software complex for tensotremometric measurements in psychophysiological research. Russ. Psychol. J. 20, 6–20. doi: 10.21702/rpj.2023.2.1

[ref5] Beranbaum S. Kouri N. Van Der Merwe N. DePierro V. K. D’Andrea W. (2023). Behavioral and biological indicators of risk and well-being in a sample of south African youth. J. Child Adolesc. Trauma 16, 163–172. doi: 10.1007/s40653-021-00426-1, PMID: 37234824 PMC10205918

[ref7] Buonsenso A. Murri A. Centorbi M. Di Martino G. Calcagno G. di Cagno A. . (2022). Psychological wellbeing and perceived fatigue in competitive athletes after SARS-CoV-2 infection 2 years after pandemic start: practical indications. J. Funct. Morphol. Kinesiol. 8:1. doi: 10.3390/jfmk8010001, PMID: 36648893 PMC9844459

[ref8] Cohen S. Kamarck T. Mermelstein R. (1983). A global measure of perceived stress. J. Health Soc. Behav. 24, 385–396. doi: 10.2307/2136404 6668417

[ref9] Davis S. L. Soistmann H. C. (2022). Child’s perceived stress: a concept analysis. J. Pediatr. Nurs. 67, 15–26. doi: 10.1016/j.pedn.2022.07.013, PMID: 35882112 PMC10167593

[ref10] Di Martino G. Centorbi M. Buonsenso A. Fiorilli G. Della Valle C. Calcagno G. . (2024). Post-traumatic stress disorder 4 years after the COVID-19 pandemic in adolescents with different levels of physical activity engagement: a repeated cross-sectional study. Int. J. Environ. Res. Public Health 21:975. doi: 10.3390/ijerph21080975, PMID: 39200586 PMC11353573

[ref11] Długosz P. (2022). Mental health disorders among students from rural areas three months after returning to school: a cross-sectional study among polish students. Youth 2, 271–278. doi: 10.3390/youth2030019

[ref12] Fiorilli G. Buonsenso A. Davola N. Di Martino G. Baralla F. Boutious S. . (2021). Stress impact of COVID-19 sports restrictions on disabled athletes. Int. J. Environ. Res. Public Health 18:12040. doi: 10.3390/ijerph182212040, PMID: 34831791 PMC8619846

[ref14] Hodge S. R. Sánchez O. Martínez Rivera C. Meshelemiah J. C. A. Almodóvar Morales M. G. Martín Cordero D. A. . (2023). Puerto Rican pre-teenagers’ physical and sedentary activities, dietary trends, and stress. Res. Soc. Develop. 12:e13512642195. doi: 10.33448/rsd-v12i6.42195

[ref15] Jones E. E. Reed M. Meyer A. H. Gaab J. Ooi Y. P. (2023). Stress, mental health and sociocultural adjustment in third culture kids: exploring the mediating roles of resilience and family functioning. Front. Psychol. 14:1093046. doi: 10.3389/fpsyg.2023.1093046, PMID: 37645063 PMC10461105

[ref16] Journault A. Beaumont E. Lupien S. J. (2023). Stress, anxiety, emotion regulation and social support in parent-child dyads prior to and during the onset of the COVID-19 pandemic. Stress. Health 39, 285–298. doi: 10.1002/smi.3183, PMID: 35849114 PMC9349815

[ref17] Journault A.-A. Cernik R. Charbonneau S. Sauvageau C. Giguère C.-É. Jamieson . (2024). Learning to embrace one’s stress: the selective effects of short videos on youth’s stress mindsets. Anxiety Stress Coping 37, 29–44. doi: 10.1080/10615806.2023.2234309, PMID: 37552634

[ref18] Kanchibhotla D. Subramanian S. (2021). Improvement in Children’s mental health and cognitive abilities with yogic breathing: a pilot study. Indian J. Youth Adolescent Health 8, 1–4. doi: 10.24321/2349.2880.202108

[ref19] Kanchibhotla D. Subramanian S. Kaushik B. (2021). Association of yogic breathing with perceived stress and conception of strengths and difficulties in teenagers. Clin. Child Psychol. Psychiatry 26, 406–417. doi: 10.1177/1359104521994633, PMID: 33588582

[ref21] Keane K. Evans R. R. Orihuela C. A. Mrug S. (2023). Teacher–student relationships, stress, and psychosocial functioning during early adolescence. Psychol. Sch. 60, 5124–5144. doi: 10.1002/pits.23020

[ref22] Kornienko D. S. Rudnova N. A. (2023). Exploring the associations between happiness, life-satisfaction, anxiety, and emotional regulation among adults during the early stage of the COViD-19 pandemic in Russia. Psychol. Russ. 16, 99–113. doi: 10.11621/pir.2023.0106, PMID: 37795214 PMC10547116

[ref23] Kornienko D. S. Rudnova N. A. Tarasova K. S. (2024). Psychometric properties of the perceived stress scale for children (PSS-C). Klin. Spec. Psihol. 13, 129–146. doi: 10.17759/cpse.2024130208

[ref24] Kuznetsova A. S. Gushchin M. V. Titova M. A. (2021). Work stress and proactive coping strategies in hospital nurses during the first wave of COVID-19 pandemic. Psikhologiya 2, 199–236. doi: 10.11621/vsp.2021.02.10

[ref25] Lynch T. Davis S. L. Johnson A. H. Gray L. Coleman E. Phillips S. R. . (2022). Definitions, theories, and measurement of stress in children. J. Pediatr. Nurs. 66, 202–212. doi: 10.1016/j.pedn.2022.07.008, PMID: 35868219 PMC10085063

[ref26] Manns A. Hamilton E. Gun K. H. Gathercoal K. (2022). Perceived stress and nonverbal intellectual abilities are differentially related to academic success in Latinx and European American rural elementary students. Contemp. Sch. Psychol. 26, 368–375. doi: 10.1007/s40688-020-00344-3

[ref27] Maratos F. A. Wood W. Cahill R. Tronco Hernández Y. A. Matos M. Gilbert P. (2024). A mixed-methods study of compassionate mind training for pupils (CMT-pupils) as a school-based wellbeing intervention. Mindfulness 15, 459–478. doi: 10.1007/s12671-024-02303-y

[ref28] Marie R. Journault A.-A. Cernik R. Welch P. Lupien S. McDermott B. . (2022). A cross-sectional study investigating Canadian and Australian adolescents’ perceived experiences of COVID-19: gender differences and mental health implications. Int. J. Environ. Res. Public Health 19:4407. doi: 10.3390/ijerph19074407, PMID: 35410086 PMC8998759

[ref29] McEwen B. S. Akil H. (2020). Revisiting the stress concept: implications for affective disorders. J. Neurosci. 40, 12–21. doi: 10.1523/JNEUROSCI.0733-19.2019, PMID: 31896560 PMC6939488

[ref30] Mochida S. Sanada M. Shao Q. Lee J. Takaoka J. Ando S. . (2021). Factors modifying children’s stress during the COVID-19 pandemic in Japan. Eur. Early Child. Educ. Res. J. 29, 51–65. doi: 10.1080/1350293X.2021.1872669

[ref31] Patil P. P. Karale R. Mohite V. R. Naregal P. (2021). Effectiveness of art therapy on level of stress and anxiety among paediatric oncology patients. Sri Lanka J. Child Health 50:459. doi: 10.4038/sljch.v50i3.9696

[ref32] Pervichko E. I. Mitina O. V. Stepanova O. B. Konyukhovskaya Y. E. Shishkova I. M. Dorokhov E. A. (2022). Perceptions of the COVID-19 pandemic and psychological distress amongst Russian citizens during spring 2020. Consort. Psychiatricum 3, 70–86. doi: 10.17816/CP136, PMID: 39045122 PMC11262101

[ref33] Rongo R. Michelotti A. Bucci R. Vitale F. Stoustrup P. Valletta R. (2024). Evaluation of psychosocial aspects in patients with juvenile idiopathic arthritis. Semin. Orthod. 30, 259–266. doi: 10.1053/j.sodo.2023.12.009

[ref34] Skinner E. A. Zimmer-Gembeck M. J. (2007). The development of coping. Ann. Rev. Psychol. 58, 119–144. doi: 10.1146/annurev.psych.58.110405.085705, PMID: 16903804

[ref35] Sobko T. Liang S. Cheng W. H. G. Tun H. M. (2020). Impact of outdoor nature-related activities on gut microbiota, fecal serotonin, and perceived stress in preschool children: the Play&Grow randomized controlled trial. Sci. Rep. 10:21993. doi: 10.1038/s41598-020-78642-2, PMID: 33319792 PMC7738543

[ref36] Takeuchi H. Napier-Raman S. Asemota O. Raman S. (2022). Identifying vulnerable children’s stress levels and coping measures during COVID-19 pandemic in Japan: a mixed method study. BMJ Paediatrics Open 6:e001310. doi: 10.1136/bmjpo-2021-001310, PMID: 36053626 PMC8889347

[ref37] Treves I. N. Li C. E. Wang K. L. Ozernov-Palchik O. Olson H. A. Gabrieli J. D. E. (2023a). Mindfulness supports emotional resilience in children during the COVID-19 pandemic. PLoS One 18:e0278501. doi: 10.1371/journal.pone.0278501, PMID: 37437077 PMC10337965

[ref38] Treves I. N. Olson H. A. Ozernov-Palchik O. Li C. E. Wang K. L. Arechiga X. M. . (2023b). At-home use of app-based mindfulness for children: a randomized active-controlled trial. Mindfulness 14, 2728–2744. doi: 10.1007/s12671-023-02231-3, PMID: 38654938 PMC11034918

[ref39] Vasanthakumari S. Vijayalakshmi S. Mayapatlia. (2016). Effectiveness of stress reduction technique on the level of stress among HIV infected children. J. Nursing Trendz 7:10. doi: 10.5958/2249-3190.2016.00003.1

[ref40] White B. P. (2014). The perceived stress scale for children: a pilot study in a sample of 153 children. Int. J. Pediatrics Child Health 2, 45–52. doi: 10.12974/2311-8687.2014.02.02.4

[ref41] Yılmaz Koğar E. Koğar H. (2024). A systematic review and meta-analytic confirmatory factor analysis of the perceived stress scale (PSS-10 and PSS-14). Stress. Health 40:e3285. doi: 10.1002/smi.3285, PMID: 37341705

[ref42] Zolotareva A. A. (2023). Psychometric properties of the Russian version of the perceived stress scale (PSS-4, 10, 14). Education 12, 18–42. doi: 10.17759/cpse.2023120102

